# Induction of hyperammonia in irradiated hepatoma cells: a recapitulation and possible explanation of the phenomenon

**DOI:** 10.1038/sj.bjc.6601915

**Published:** 2004-06-08

**Authors:** J van Rijn, J van den Berg, R G Schipper, S de Jong, V Cuijpers, A A J Verhofstad, T Teerlink

**Affiliations:** 1 Radiation Oncology, VU University Medical Center, PO Box 7057, 1007 MB Amsterdam, The Netherlands; 2 Metabolic Unit, Department of Clinical Chemistry, VU University Medical Center, Amsterdam, The Netherlands; 3 Department of Pathology, University of Nijmegen Medical Center, Nijmegen, The Netherlands

We recently reported that a high amount of ammonia was produced in the culture medium of H35 hepatoma cells in reponse to ionising radiation (Van Rijn *et al*, 2003). As a result of hyperammonia and the accompanying pH increase, surviving cells failed to develop into colonies at high inoculation densities. The expected clonogenic potential of the cells reverted to more normal levels when the culture medium of irradiated cultures was replaced by a fresh medium halfway through the postexposure period. Even so, colonies were reduced in size. The removed medium proved cytotoxic to nonirradiated cells, indicating that irradiation *per se* was not responsible. Analysis of the medium implicated ammonia as the primary cytotoxic compound. Endogenously produced ammonia gas acts like a free radical, and is very aggressive and cytotoxic until it is converted into ammonium ions, which cells can tolerate at millimolar levels. Since native ammonia produces alkalinity, raised pH also accounts for cell death. Parallel with the development of hyperammonia, ornithine and citrulline levels were raised, whereas arginine was lost from the medium. Thus, this minimal deviation hepatoma seemed to be able to convert ammonia into urea through a fully functional ornithine cycle. This was thrown in doubt when it was established that the cells did not proliferate when ornithine was added in place of arginine. Replacement of arginine by citrulline, on the other hand, worked well and also prevented the development of hyperammonia (Van Rijn *et al*, 2003).

The accumulation of ornithine in the medium and a report on the induction of ornithine decarboxylase by ionising radiation (Grassilli *et al*, 1995) pointed to the possibility that polyamines were involved in the formation of ammonia. Furthermore, since the medium toxicity was not fully explained by the presence of ammonia and cell death appeared to be apoptotic (Van Rijn *et al*, 2003), the idea was advanced that the cytotoxicity was partly due to disturbance of polyamine homeostasis (Schipper *et al*, 2000). An excess of polyamines then could be a potential source for the ammonia (Seiler, 2000).

Investigation of the synthesis and interconversion pathways of the diamine, putrescine, and the polyamines, spermidine and spermine was undertaken. Several inhibitors are known to interfere with the key enzymes, ornithine decarboxylase and *S*-adenosylmethionine decarboxylase, or the diamine- and polyamine oxidases. Many of the inhibitors, for example, *α*-difluoromethylornithine, L-agmatine, dicyclohexylamine, methylglyoxal-*bis*-guanylhydrazone and CGP48664A, reduced the release of ammonia. Conversely, the addition of the depleted product (putrescine, spermidine or spermine) or of inhibitors of the diamine/polyamine oxidases (aminoguanidine and MDL72527) restored hyperammonia. However, any suppression of the hyperammonia development was accompanied by a decreased conversion of arginine into ornithine and citrulline. Furthermore, most of the inhibitors except L-agmatine reduced the clonogenic survival in the combination with X-rays. It was therefore concluded that the ammonia reduction was possibly due to cytotoxicity of these drugs.

These studies generated a large amount of data on the ornithine cycle compounds. Analysis showed that the sum of the molar concentrations of arginine, ornithine and citrulline did not change, but remained at ∼2.5 mM, which is the amount of arginine present in the fresh L15 medium (Table 1

**Table 1:** Effect of X-rays on the formation of ammonia in cell cultures

	**Cells × 10^–3^**	**Ammonia**	**Arginine**	**Ornithine**	**Citrulline**
*H35 mp+*					
8 Gy X-rays	62	4.52	0.32	1.45	0.80
Control	5282	2.49	1.54	0.63	0.29
Norvaline	2999	1.55	2.55	0.09	0.06
					
*H35 mp−*					
8 Gy X-rays	78	0.89	2.35	0.21	0.023
Control	3891	1.52	2.32	0.20	0.024
Norvaline	1976	1.48	2.40	0.08	0.026
					
*D384 mp+*					
8 Gy X-rays	375	4.87	Nil	1.04	1.31
Control	2002	4.58	Nil	1.01	1.39
					
*D384 mp−*					
8 Gy X-rays	428	1.14	2.38	0.19	0.023
Control	2195	1.41	—	—	—
					
Medium only		1.10	2.43	0.19	0.023

Mycoplasma-contaminated and mycoplasma-free cell cultures were investigated as described earlier (Van Rijn *et al*, 2003). Concentrations are in mM. The incubation time was 96 h starting with either 50 000 (H35) or 200 000 cells (D384).

). Thus, polyamines could not be implicated in the development of hyperammonia. Since it was found that cells did not proliferate in the medium in which arginine was replaced by ornithine, it was suspected that the conversion of ornithine into citrulline probably occurs too slowly to provide for an adequate supply of arginine. Although when the citrulline was found not to come from ornithine, it was clear that a different pathway had to be involved.

A review of the literature revealed that an enzyme exists, arginine deiminase (EC 3.5.3.6), that converts arginine directly into citrulline and ammonia. In the reverse direction, aspartate is used as an amino donor for the synthesis of arginine, that is, as part of the urea cycle, and involves argininosuccinate synthase and argininosuccinate lyase. Since the ammonia/citrulline ratio is >4, this means that a repeated cycling has to be executed between arginine and citrulline in order to create hyperammonia. The possibility that this cultured cell line contained arginine deiminase activity pointed to a different source, that is, contamination with mycoplasma (Miyazaki *et al*, 1990). When this was tested, the hepatoma cell line proved mycoplasma positive. The type of mycoplasma has still to be ascertained and the presence of arginine deiminase itself verified. However, the cell cultures were treated with MycoKill™ over 3 weeks and retested. They were now mycoplasma negative. The human glioma cell line, D384, which also develops hyperammonia received the same treatment and was freed of mycoplasma contamination.

Both mycoplasma-free cell lines were exposed to X-rays in order to see whether they would now produce hyperammonia, but the ammonia levels remained below that of the proliferating control cells (Table 1). Furthermore, amino-acid analysis showed that extensive breakdown of arginine did not occur in the decontaminated cell lines, and that neither citrulline nor ornithine levels were influenced by the presence of the cells.

The apparent coupling between the presumed activities of arginine deiminase and arginase in mycoplasma-infected cultures is interesting when one considers the question about the origin of the arginase activity. When it is derived from the mycoplasma, the answer is clear. Otherwise, when it is of hepatic origin, it might be assumed that the products of the presumed mycoplasmic arginine deiminase cause the activation of the hepatic arginase.

In an attempt to test this experimentally, mycoplasma-free H35 cells were incubated with citrulline, ammonia or a combination. Neither citrulline (2 mM) nor the presence of 4 mM ammonium chloride had an effect on the ornithine concentration. In arginine-deficient medium with citrulline, the formation of ornithine decreased by almost 80%, which indicates that arginine is the primary source for the ornithine. It was also found that the medium only accounted for the increase in ornithine, since it was the same in the presence or absence of the cells.

In conclusion, H35 cells do not contain significant (detectable) levels of arginase activity that affects the composition of the culture medium. As for the mycoplasma-positive cells, both the assumed enzyme activities, arginase and arginine deiminase, appear to originate from the mycoplasma. In addition, a little arginase activity was suspected in the serum of the culture medium, since it showed sensitivity to the inhibitor, L-norvaline (Table 1).

In our previous report, we showed that the production of ammonia and the accompanying increases in ornithine and citrulline were significantly reduced by L-norvaline (Van Rijn *et al*, 2003). A similar result was obtained with L-agmatine, a natural derivative of arginine, although at a much lower dose of 0.2 mM (results not shown). This indicates that these compounds can inhibit both arginase and arginine deiminase. The suggestion that L-agmatine is a potent inhibitor of these enzymes, together with its effects on NO synthase and ornithine decarboxylase (Cabella *et al*, 2001), makes it a very interesting substance worthy of much greater investigation in the regulation of the arginine metabolism and of the synthesis of diamines and polyamines.

Finally, and in contrast to mycoplasma-contaminated cells, L-norvaline (20 mM) did not influence the production of ammonia in the ‘cured’ cell cultures, indicating that it originates from a different (cellular) source (Table 1).

In conclusion, the development of hyperammonia in H35 cultures in response to X-rays appears to be caused by arginine deiminase due to an infection with mycoplasma. In proliferating cell cultures, the phenomenon is probably suppressed during the exponential growth, but it appears to become manifest when cell growth is restricted, that is, in confluent cultures. In a similar way, this might happen in growth-restricted low-density populations like after a high dose of X-rays, which then probably does not affect the mycoplasma growth to the same extent. As the irradiated cells are not killed outright, they might support mycoplasma growth by providing necessary growth factors. Also the mycoplasma might be more resistant to X-rays. The inhibition of the ammonia production with the various inhibitors of the polyamine metabolism could possibly be attributed to an inhibition of mycoplasma growth. The suppression of hyperammonia development with norvaline or agmatine probably reflects an inhibition of arginine deiminase, but this requires substantiation. The reduction of hyperammonia in the medium with citrulline instead of arginine is probably because the synthesis of arginine from citrulline is rate limiting. The data overall lead us to the conclusion that mycoplasma infection might go unnoticed, being ‘relatively harmless’ until cells are exposed to specific experimental conditions or stressed in some way that allows them to express themselves when cell proliferation is compromised.
